# Fabrication and Characterization of Nylon 66/PAN Nanofibrous Film Used as Separator of Lithium-Ion Battery

**DOI:** 10.3390/polym13121984

**Published:** 2021-06-17

**Authors:** Yu-Hsun Nien, Chih-Ning Chang, Pao-Lin Chuang, Chun-Han Hsu, Jun-Lun Liao, Chen-Kai Lee

**Affiliations:** 1 Department of Chemaical and Materials Engineering, National Yunlin University of Science and Technology, Yunlin 640, Taiwan; m10615007@gemail.yuntech.edu.tw (C.-N.C.); chuangpl@yuntech.edu.tw (P.-L.C.); m10815014@gemail.yuntech.edu.tw (J.-L.L.); peter862525@gmail.com (C.-K.L.); 2 General Education Center, National Tainan Junior College of Nursing, Tainan City 700, Taiwan; chunhanhsu@gmail.com

**Keywords:** electrospun, separator, lithium-ion battery, nylon 66, polyacrylonitrile

## Abstract

In recent years, portable electronic devices have flourished, and the safety of lithium batteries has received increasing attention. In this study, nanofibers were prepared by electrospinning using different ratios of nylon 66/polyacrylonitrile (PAN), and their properties were studied and compared with commercial PP separators. The experimental results show that the addition of PAN in nylon 66/PAN nanofibrous film used as separator of lithium-ion battery can enhance the porosity up to 85%. There is also no significant shrinkage in the shrinkage test, and the thermal dimensional stability is good. When the Li/LiFePO_4_ lithium battery is prepared by nylon 66/PAN nanofibrous film used as separator, the capacitor can be maintained at 140 mAhg^−1^ after 20 cycles at 0.1 C, and the coulombic efficiency is still maintained at 99%, which has excellent electrochemical performance.

## 1. Introduction

In the twentieth century, the number of miniaturized devices increases as microelectronics technology develops, and there are more requirements for power supplies. Lithium batteries have entered a stage where they have been put into practical use on a large scale, especially in electronic handheld devices. For example, smart phones, notebook computers, and cameras all have lithium batteries. The main work of lithium battery is to transform the chemical energy into electrical energy, and to use the electrons produced by the electric pole to circulate the electricity flow in the electrolyte. The pore distribution, thickness, and the mechanical properties of the separator affect the battery life, electricity capacity, and safety. Therefore, in the battery design, the separator has the most important role regarding its safety.

However, in recent years, there have been numerous incidents concerning the expansion and explosion of mobile phone batteries, making the safety of lithium batteries as one of the most eye-catching projects. The four key materials of the lithium battery are cathode material, anode material, separator, and electrolyte. The main function of the separator is to separate the positive and negative electrodes to prevent short-circuit caused by contact between the two poles. At the same time [1], it has good electrolyte conductivity, and the performance of the separator directly affects the efficiency and safety of the battery. Therefore, improving the performance of the separator is one of the most important studies at present.

Due to the requirement of chemical stability and electrolyte absorptivity for lithium battery isolation membranes, the development of raw materials has gradually changed from nonwoven fibers to polypropylene (PP) and polyethylene (PE) fibers in polyolefin systems. However, polyolefin materials shrink sharply at high temperature, making lithium batteries unsafe. Therefore, many studies have been carried out to improve materials and processes with safety as the main objective. High performance separators must (1) be porous, (2) be safe against puncture and shorts, (3) have high melting stability, (4) have a thin structure for more active material, (5) have a very thin layer for composite structures of ceramic coatings, (6) be of very thin and porous nonwoven fibers for filling with polymer electrolyte, (7) have high thermal stability, and (8) be low cost for use in hybrid vehicles [2].

Electrospinning technology is a continuous nano-sized polymer fiber technology generated by applying an external electric field to a polymer solution [3]. The parameters of the control process in the electrospinning process are divided into three parts: solution parameters, process parameters, and ambient parameters. The above parameters directly affect the fiber morphology. Through the control of the parameters, the optimal state can be found and then a good fiber film can be produced [4,5,6]. In recent years, many studies have pointed out that the electrospun fiber membrane has high porosity and its unique pore structure can be used as a lithium battery separator [7,8].

Cho et al. published a study on polyacrylonitrile (PAN) electrospun nanofibers, which were applied to create separators and several experiments were performed [9]. The results indicate that the PAN separator has a lower contact angle than the Celgard 2400 commercial separator, which indicates the higher wettability of PAN separator. Lithium battery charging and discharging tests using PAN separator have been shown to be as stable as commercial separators, while charge–discharge cycling testing using PAN separator has shown better discharge capacity performance than when using commercial separators. Commercial separators and PAN separators are fabricated into lithium batteries and charged to 4.2 V. After 1 h at 120 °C; the two separators do not change. When the temperature is raised to 150 °C, the voltage dropped after 10 and 14 min, respectively. The reason is that the separators shrink in the presence of electrolyte and cause short circuit in the battery. Therefore, the thermal stability of the PAN separator is not significantly better than that of a commercial separator. Evans et al. also report PAN nanofiber separators [10]. The tensile strength of PAN is 16.39 MPa, which is lower than 114 MPa of the Celgard PP separator. The SEM test shows that the PAN nanofibers are swollen by the electrolyte after 100 cycles of testing. Rao et al. report a gel polymer electrolyte (GPE) of PAN/PMMA by the electrospinning method [11]. PAN and PMMA are first dissolved in DMF in a ratio of 4:1, and then a nanofiber film is formed by electrospinning, and then the film is immersed in 1 M LiTFSi to prepare a gel polymer electrolyte. The electrolyte uptake of the GPE is 480%, while the electrolyte uptake of PAN nanofibers is 420%, and the commercially available separator Celgard PE is only 120%. Charge and discharge performance tests and electrochemical tests also show that GPE prepared by PAN/PMMA is more stable and better than PAN and Celgard PE. Lee et al. also report PAN nanofiber separators [12]. They indicate that the cyclization and oxidation reaction are the main reactions during the heat treatment of PAN. Both reactions are important to form ladder polymer structure, which is thermally stable and able to resist high temperature during carbonization reaction for the preparation of carbon fibers. PAN-C membrane is heat-treated at 230 °C to improve thermal stability and tensile strength.

In a study by Guerini et al., different molecular weights of nylon 66 are dissolved in formic acid for electrospinning [13], and it is found that nanofibers cannot be produced when the molecular weight is lower than *M*_w_ = 27,759 gmol^−1^. The average diameter of nanofibers increases with increasing molecular weight. Wu et al. study PA66 electrospinning with formic acid to form 15 wt.% solution. They collect nanofibers with an electrode rod of 0.5 mm in diameter [14]. It is found that the nanofibers have better directivity when the collecting rods are not rotated. When the collecting rods are rotated at 300 rpm, the nanofibers are irregularly arranged. The tensile test shows that the tensile strength of the nanofibers collected at a speed of 300 rpm is 30.3 MPa. When the collecting rods are not rotated, the nanofibers have a tensile strength of only 11.7 MPa. Zussman uses a roller collector to collect PA66 nanofibers [15]. When the collecting roller speed is 20 ms^−1^, the average diameter of PA66 nanofiber is smaller than the roller speed of 5 m/s, and its Young’s modulus and strength become higher. Pant performs the electrospinning studies on continuous dissolution of nylon 6 by dissolving formic acid and acetic acid in a solvent ratio of 4:1, and then electrospinning at 12 kV, 22 kV, and 32 kV, respectively [16]. When the voltage is 22 kV, high-directional nanofibers are formed, and the fiber surface is smooth with a spider-like structure. The spider-like network is connected between the main fibers, and its formation is mainly due to the hydrogen bonding of nylon 6. Matulevicius et al. study the structure of nylon 66 spider-like fibers using 2D and 3D AFM [17]. The individual fibers have a width of 465 nm and a height of 220 nm, and the spider-like network fibers have a width of 9 to 28 nm and a height of 7 to 15 nm.

Due to its high surface area/volume ratio and high porosity, electrospun nanofibers can be used in lithium-ion batteries’ separators. High porosity allows the separator to have more ion-permeable channels and high electrolyte uptake. The electrospun nanofiber separator has better electrical conductivity than those of commercial PE/PP separators. However, due to its high porosity, its mechanical properties are lower than those of commercial PE/PP separators. In order to develop separators with high mechanical properties and high temperature resistance, nylon 66 was used as the main material for those separators in this study, and polyacrylonitrile was added to produce nylon 66/PAN separators by high-voltage electrospinning. The nanofiber film produced by the electrospinning method has a high specific surface area, and its porosity can be as high as 80%, which is much higher than that of the conventional extrusion stretched polyolefin film (<50%). Due to its high porosity, it may be coated or impregnated by inorganic materials in the future, which further enhances its thermal stability, wettability to the electrolyte, and mechanical strength. In the study, we know that adding the right amount of PAN layer between two nylon 66 layers can actually improve battery performance, especially in porosity, electrolyte absorption rate, thermal stability, and the capacitance. The most important thing is that the separator has a shut-off mechanism. When the battery temperature is higher than the Tg of PAN, the PAN begins to soften to act as a barrier within nylon 66 nanofibers to prevent the electrode contact, which causes a short circuit.

## 2. Materials and Methods

### 2.1. Materials

Nylon 66 (*M*_w_ = 262.35) was purchased from Sigma-Aldrich (St. Louis, MA, USA). Formic acid (HCOOH) was provided by Katayama Chemical (Osaka, Japan) with a purity of 98–100%. Polyacrylonitrile (PAN, *M*_w_ = 150,000) was from Scientific Polymer Products, Inc (Ontario, NY, USA). Dimethyl sulfoxide (DMSO, *M*_w_ = 78.13) was supplied from PanReac AppliChem (Chicago, IL, USA) with a purity of 99.5%. All the other chemicals used were analytical grade. Liquid electrolyte, the EC solvent (battery grade, extra dry < 20 ppm of water), was purchased from Ferro Corp (Mayfield Heights, OH, USA) and used as received without further purification. The commercialized PP separator (Celgard 2320), provided by Celgard company (Charlotte, NC, USA) was regarded as the separator of Li-ion batteries for comparison.

### 2.2. Preparation of Nylon66 and PAN Electrospinning Solution and Separators

Research on nylon 66 for high-voltage electrospinning shows that the use of formic acid as a solvent can produce a spider-like structure, which can enhance its mechanical strength [11,12]. In this study, nylon pellets were weighed and dissolved in formic acid and placed in a sample vial to prepare a solution with a concentration of 10%. The mixture was mechanically stirred for 12 h until the nylon particles were completely dissolved and the solution appeared in a transparent state.

PAN is able to dissolve in DMF and DMSO. DMF is highly dangerous to humans. Therefore, this study selected DMSO with relatively low risk as the solvent for this experiment. The PAN at a concentration of 8 wt.% was prepared by adding PAN into DMSO and mechanically stirred for 12 h. PAN solution should not be put out for a long time, and should be used as soon as possible after preparation.

**Scheme 1:**
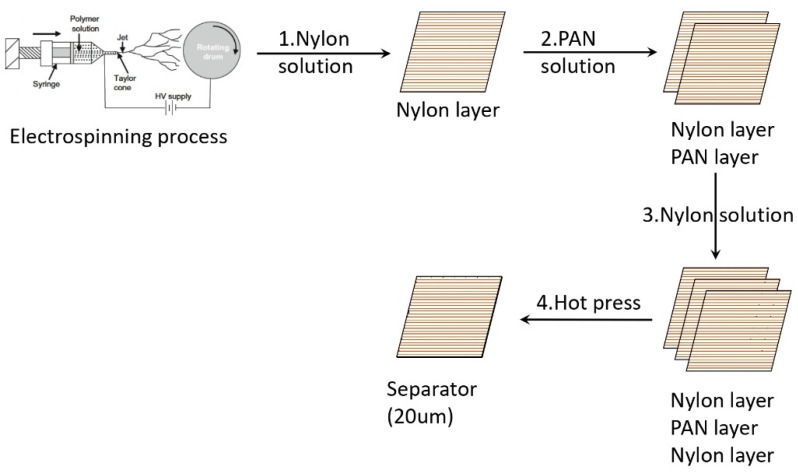
The separator production flow chart.

The first layer separator was stretched by electrospinning 4.5 mL of the prepared nylon solution at a flow rate of 0.007 mL min^−1^. The ratio of the PAN solution was 1.25, 2.5, and 3.75 mL, respectively, and the flow rate was 0.017 mL min^−1^, and the solution was prepared as a second layer separator. Finally, 4.5 mL of the nylon 66 solution was extracted as a third layer separator at a rate of 0.007 mL min^−1^. Our production method is shown in Scheme 1. It can be seen that we perform hot pressing after electrospinning.

### 2.3. Hot-Pressing Procedure of Nylon 66/PAN/Nylon 66 Nanofibers

The electrospun nanofibers were cut into squares having a width of 10 cm and a length of 10 cm, and the three layers of electrospun fibers were hot pressed. The hot-pressing conditions were 155 °C and the pressure was 300 psi for 30 min.

In this study, three kinds of nanofiber separators were prepared by electrospinning technology, and different ratios of nylon 66/PAN were added, respectively, in weight ratios of 9:1:9, 9:2:9, and 9:3:9. For ease of description and discussion, Table 1 represents the list of symbols and their representative meanings.

### 2.4. Measurement System

The sample was cut into the appropriately sized test strips then attached to a conductive disc by a conductive tape and sputtered with platinum. The surface structures of the samples were observed using FE-SEM (JEOL JSM-7000F) at the voltage of 15 kV and a magnification of 10,000 times.

The thermal scanning (DSC, PerkinElmer DSC-6000) mode was ranged from 30 to 800 °C at a programming heating rate of 20 °C min^−1^ in nitrogen with a gas flow of 20 mL min^−1^. The thermal stabilities of the four high performance fibers were studied by thermogravimetric.

The program temperature was set from room temperature to 350 °C and the heating rate was 10 °C min^−1^, and the flow rate of the gas was 20 mL min^−1^. The ratio of the theoretical 100% crystallinity ΔHf to ΔHfs (ΔHf of sample) is the crystallinity, and the formula is as shown in Equation (1) [13], nylon 66 Δ*H*_f_ = 255.4 J g^−1^ [18].

(1) $$\mathrm{Crystallinity}\;(\%)={\Delta H}_{\mathrm{fs}}{\Delta H}_{\mathrm{fs}}{}^{-1}\times 100\%$$

The sample was cut into a strip shape of 10 mm × 50 mm and the tensile strength and elongation of the samples were measured using a tensile tester (Hung Ta HT-2402EC) at a rate of 10 mm min^−1^.

The dry sample was immersed in propylene glycol for one minute and the test piece was observed to be translucent, and dried to a mark of no water stain to measure the weight of the wet sample. The porosity was calculated by using the following Equation (2) [19,20,21].

(2) $$\mathrm{Porosity}\;(\%)={(w}_{w}-w_{d})\rho^{-1}V^{-1}\times 100\%$$

where $w_{w}$ and $w_{d}$ are the weights of wet and dry membranes, respectively, ρ is the density of propylene glycol, and V is the membrane geometric volume.

In order to ensure that the positive and negative electrodes of the battery do not contact each other, a dimensional heat shrinkage test was performed. The shrinkage rate was calculated by using Equation (3) [7,8,9].

(3) $$\mathrm{Shrinkage}\;(\%)=100-A_{a}A_{b}{}^{-1}\times 100$$

where $A_{b}$ and $A_{a}$ are the areas before and after heating of the separator, respectively.

The electrolyte uptake rate test was performed by cutting the separator test piece into a circular shape of 10.67 cm^2^ and immersing it in an electrolytic solution. Allow to stand at room temperature for two hours. Remove the test piece and wipe off the excess electrolyte to weigh the weight, and then calculate it using Equation (4) [22].

(4) $$\mathrm{Electrolyte}\;\mathrm{uptake}\;(\%)={(w}_{w}-w_{d})w_{d}{}^{-1}\times 100\%$$

where $w_{w}$ and $w_{d}$ are the weights of wet and dry membranes, respectively.

Lithium iron phosphate was used as the cathode material of the lithium-ion battery, and the lithium foil was used as the anode. The anode material, the separator, and the cathode material were sequentially placed in the CR2032 battery case, and then the electrolyte was added. Subsequently, the battery cover was covered and the package was pressed together. The CR2032 button battery was used for lithium-ion battery charge and discharge cycle test (Battery Automatic Tester, AcuTech Systems BAT-750B).

## 3. Results

### 3.1. Separator Morphology

**Figure 1:**
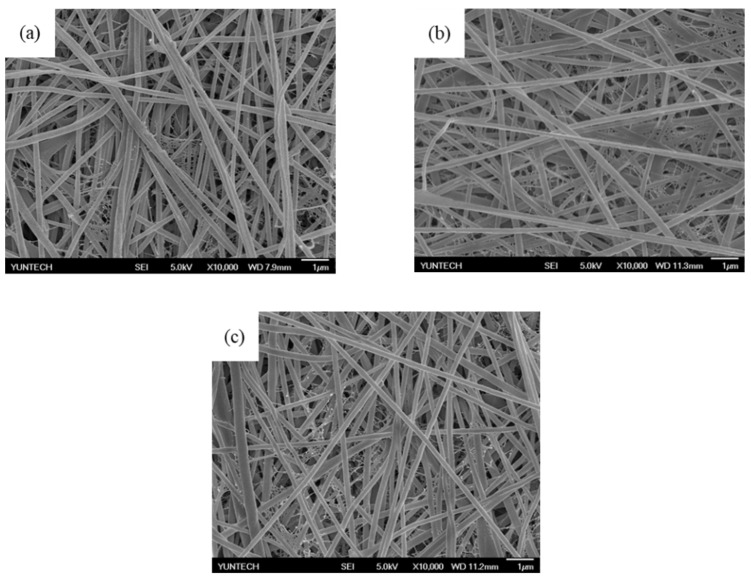
SEM image of NPN sample (**a**) NPN 919 (**b**) NPN 929 (**c**) NPN 939, the magnification is 10,000 times.

We studied the fiber surface morphology of NPN separators by SEM. The results of the NPN919, NPN929, and NPN939 samples are shown in Figure 1. Figure 1 shows that the separators of the present experiment have a fibrous structure similar to a spider web. Wang et al. have mentioned in the literature [23] that the generation of spider webs in electrospinning can increase the specific surface area of the membrane and improve surface activity. Secondly, it is possible to increase the porous structure, promote the electrolyte transport of the membrane, reduce the impedance, and enhance the electrochemical properties. The SEM surface morphology shows that the separator of this study is porous.

### 3.2. TGA Analysis

**Figure 2:**
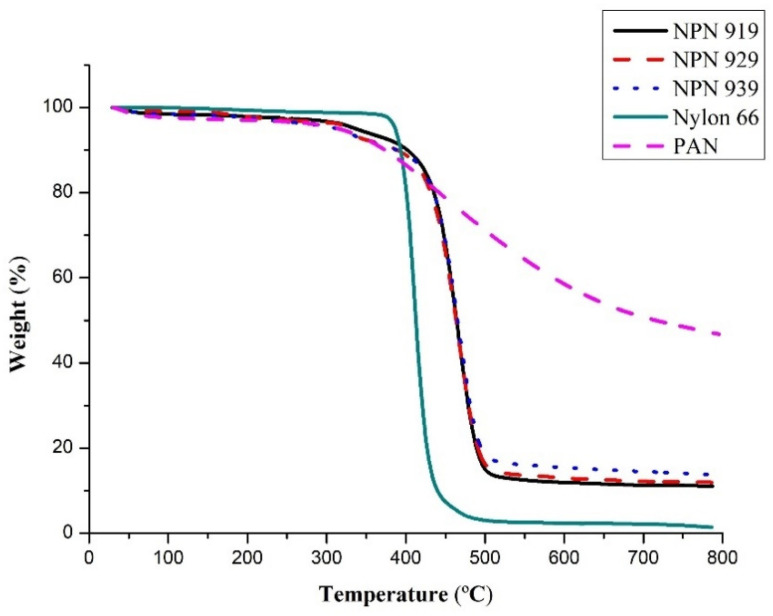
The TGA curves of nylon 66, PAN, and different NPN samples.

The nylon 66, PAN, and NPN samples are compared and analyzed by TGA, as shown in Figure 2. Figure 2 also shows that the thermal cracking curves of the respective samples are similarly overlapped, and both have a solvent or a thermal weight loss of about 2 wt.% at a temperature of about 70 °C. Table 2 lists the 10% weight loss temperatures (T10) of the nylon 66, PAN, and NPN samples. It can be seen from Table 2 that the 10% weight loss temperature of the original PAN is 373.81 °C. The temperatures of the NPN samples obtained by the electrospinning procedure are 401.90, 389.10, and 388.97 °C, respectively. Comparing all of them, the 10% weight loss temperature increased about 10–20 °C after modification. This is attributed to the fact that the electrospinning process causes the linear alignment and packing of the polymer chains in the nanofiber separator, which is better than the original nylon 66 [24]. In addition, the fibers are bonded after the hot-pressing process, and the heat resistance of the separator is also increased.

### 3.3. DSC Analysis

**Figure 3:**
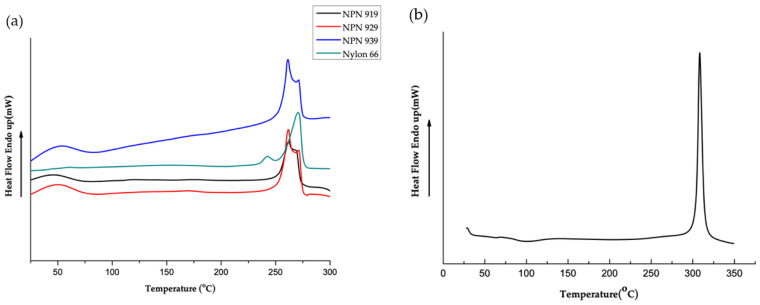
The DSC curves of (**a**) nylon 66, and different NPN samples; (**b**) nanofibers PAN.

The NPN samples are separately subjected to DSC detection, and the results are shown in Figure 3a. The DSC curve of each sample shows two distinct melting peaks. The main reason is that Nylon66 is a crystalline material, and so that nylon 66 has two melting peaks on the DSC curve, *T*_m2_ and *T*_m1_, respectively, as shown in Table 3. Bell et al. find that nylon 66 melts depending on its cooling and annealing procedures. Nylon 66 may form a melting point or two melting points [25]. Among them, *T*_m1_ is fixed. *T*_m2_ changes and may be higher or lower than *T*_m1_. *T*_m1_ can be formed via rapid cooling of molten nylon 66. The crystal melting point formed by it does not change. *T*_m2_ affects its melting point due to the cooling or annealing procedures. Bell et al. have also pointed out that the *T*_m1_ and *T*_m2_ types are produced by stretching the fibers [25]. PAN is a semi-crystalline polymer with melting point about 308 °C. The inflection point is the glass transition temperature (about 66 °C) of PAN nanofibers, as shown in Figure 3b. This result is the same as that of other studies [26,27]. The *T*_g_ of DSC curve of the NPN sample can be confirmed by the addition of PAN.

### 3.4. Mechanical Properties of NPN Separator

As shown in Table 4, the tensile strength of the nylon 66 separator was 62.64 MPa. The best tensile strength of the NPN sample is NPN919, and its tensile strength is 48.86 MPa. The trend is that the more the PAN is added, the lower the tensile strength. It is concluded that the mechanical properties of the PAN material itself are inferior to those of the nylon 66, so the more PAN added, the lower the strength, and the results are similar to those the literature [15,28]. However, Evans et al. have mentioned in the literature that the specification for the tensile strength of the separator needs to be greater than 6.89 MPa [10]. The tensile strength of the NPN separator prepared in this study is much higher than 6.89 MPa, so that the NPN sample still has very good mechanical properties.

### 3.5. Mechanical Properties of NPN Separator

**Figure 4:**
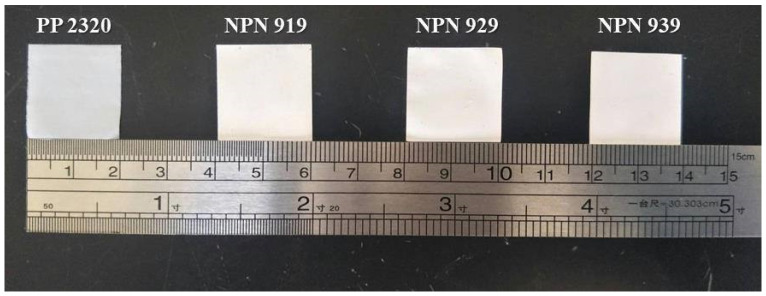
Separator samples before heating.

**Figure 5:**
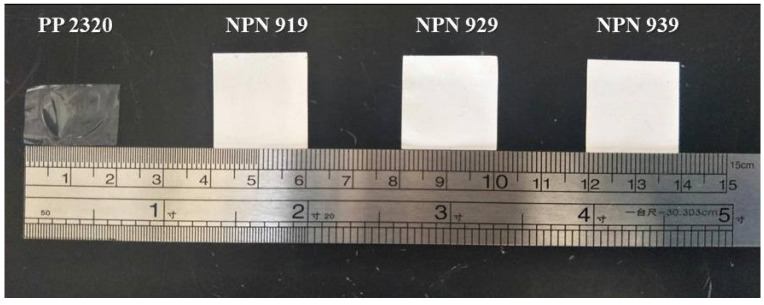
Separator samples after heating.

The samples were cut to a size of 2 cm × 2 cm, as shown in Figure 4. They were placed in a high temperature furnace with temperature at 160 °C for 15 min, and as shown in Figure 5. The test results are shown in Table 5. The shrinkage rate of the commercial PP separator after heating is 39.76%, and the shrinkage of different NPN samples are between 0.25% and 1.7%. It can be reduced to 5% according to the horizontal and vertical directions shown in USABC [20]. Therefore, the NPN sample can be proved to be a good and safe separator.

### 3.6. Separator Porosity and Electrolyte Uptake Rate

Table 6 lists the porosity of the samples and commercial separator. The commercial PP separator has a porosity of only 41.78%, while the porosity of the NPN separator is much larger than that of a commercial PP separator. Among them, the porosity of NPN 939 is 85.85%, and the minimum porosity of NPN 919 is 81.51%. The trend increases with the increase in PAN, and it is mentioned in the literature. Because of the high porosity of PAN itself, the addition of PAN makes the porosity of the overall separator increase [12].

The electrolyte absorption rate test results are shown in Table 7. The trend is the same as the porosity, which increases with the increase in PAN content. The highest NPN 939 is 405.56%, and the lowest NPN 919 is 318.18%. It can be seen that the porosity is closely related to the electrolyte intake rate.

### 3.7. Property of Charge and Discharge

**Figure 6:**
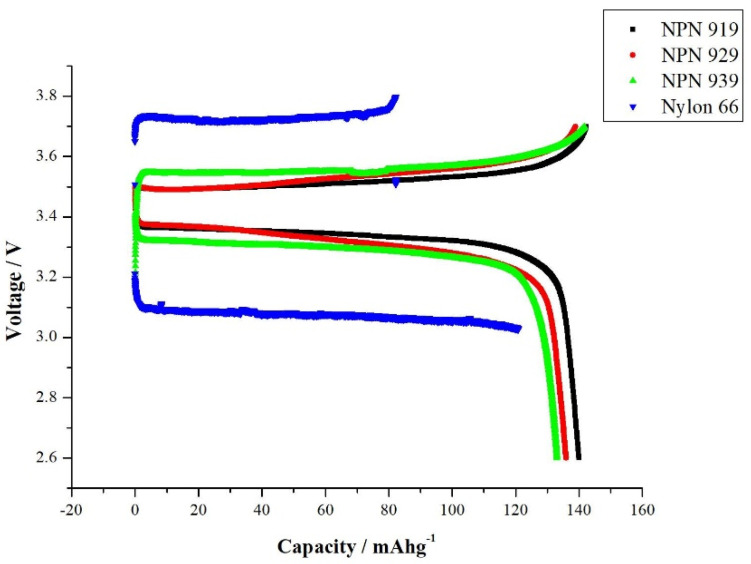
The first-cycle charge–discharge curves of Li/LiFePO_4_ cells with different NPN and nylon 66 separators, respectively.

The first charge–discharge curve of a Li/LiFePO_4_ battery at a current density of 0.1 C is shown in Figure 6. The NPN sample is compared with the nylon 66 separator, wherein the voltage difference between the charge and discharge curves is proportional to the ionic and electronic conductivities of the battery. In Figure 6, after adding PAN, the voltage difference between the charging and discharging curves becomes smaller. This means that adding PAN increases the ionic conductivity of the battery. The capacitance of the battery is shown in Table 8. It shows that nylon 66/PAN nanofibrous film used as separator has higher capacitance than the nylon66 separator, and the efficiency of the lithium battery can be improved by the addition of PAN. This is because the addition of PAN can increase compatibility with the ionic electrolyte and increase the absorption of the electrolyte.

### 3.8. Cycling Performance

**Figure 7:**
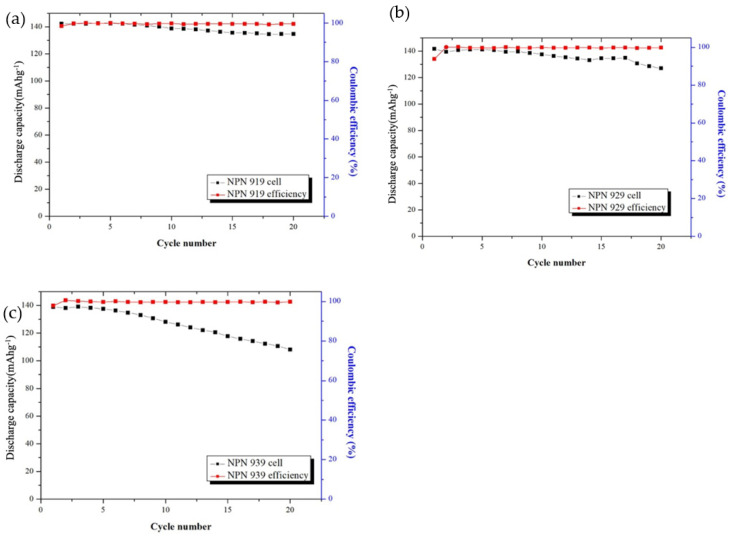
The discharge capacity and coulombic efficiency of cells with (**a**) NPN 919, (**b**) NPN 929, (**c**) NPN 939.

To investigate the electrochemical stability of the NPN sample, a battery cycle performance test was performed at 0.1 C in the range of 2.6 to 3.7 V, as shown in Figure 7. The figure shows that the discharge capacity of the NPN sample is stable, and it can be seen that the discharge capacity of all the isolation films has not decreased. The coulombic efficiency is above 99%, especially NPN 919, which is in line with the ideal state of the general lithium battery. However, with the increase in PAN, the capacitance of NPN 929 and NPN 939 gradually decreases. It is possible that the PAN fibers swell due to the absorption of the electrolyte, causing the electrolyte channel to shrink or block, resulting in a gradual decrease in capacitance. This is similar in the PVDF-PAN electrospun isolators studied by Gopalan et al. [27] and the GPE studies of PVDF/PMMA by Jung et al. [29]. Therefore, it can be proved by the experiments that the addition of PAN can actually increase the charge–discharge performance of the battery; however, if too much PAN is added, the swelling phenomenon occurs, and the battery efficiency is lowered.

### 3.9. Comparison of Commercially Available Batteries

**Figure 8:**
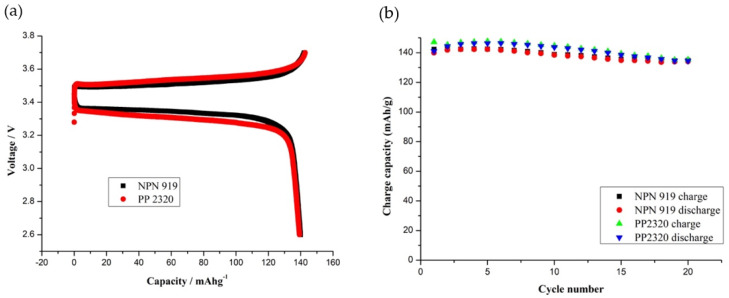
The initial charge–discharge curves (**a**) and cycling performance (**b**) of Li/LiFePO_4_ cells with PP2320 and NPN919 separators.

In order to further understand the efficiency of the NPN sample of this study, the most excellent sample NPN919 of this study was compared with the commercial PP separator for the charge–discharge test and cycle life. The result is shown in Figure 8. It shows that the capacitance and cycle life of the sample NPN919 are comparable to PP. However, the excellent thermal stability of the NPN sample, as shown in Table 4, makes the NPN sample of this study more competitive than the commercial PP separator.

## 4. Conclusions

In this experiment, nylon66/PAN/nylon66 (NPN) three-layer nanofiber separator is successfully prepared by electrospinning technology. The porosity of the separator produced in the study is about 80–85%, which is higher than that of the Celgard PP separator. The electrolyte absorption rate of the NPN electrospun nanofibrous separator increases from 318 to 405%, which is much higher than that of the Celgard PP separator. The tensile strength of the separator produced in this study is about 33–50 MPa. Although it is lower than that of the Celgard PP separator (150 MPa), it is much higher than that of the electrospun separator of PVDF, PAN, or other materials. However, the nylon 66/PAN separator in this study is significantly better than the Celgard PP separators in heat shrinkage property. It shows that nylon 66/PAN separator is more suitable for lithium-ion battery in thermal stability and safety. It is more suitable when used in high temperature equipment.

By assembling these separators into cells for electrochemical performance testing, the results show that the NPN separator has higher ionic and electronic conductivities than nylon66 separator, which can improve the efficiency of lithium battery. Adding the right amount of PAN layer between two nylon 66 layers can actually improve battery performance. Compared to a commercial PP separator, its capacity and charge–discharge cycle life are equivalent. However, NPN 919 separator has high porosity, high electrolyte absorption rate, good mechanical properties, and excellent thermal stability. The NPN 919 separator of this study is more competitive than the commercial PP separator.

## Figures and Tables

**Table 1:** List of symbols and their representative meanings.

Symbol	Representative Meaning
N:P:N	nylon 66: PAN: nylon 66
PP2320	Commercial PP microporous membrane
NPN919	Weight ratio of nylon 66: PAN: nylon 66 → 9:1:9
NPN929	Weight ratio of nylon 66: PAN: nylon 66 → 9:2:9
NPN939	Weight ratio of nylon 66: PAN: nylon 66 → 9:3:9

**Table 2:** The 10% weight loss temperature (T10) of nylon 66, PAN, and different NPN samples.

Separator	10% Weight Loss Temperature T10 (°C)
NPN919	401.90
NPN929	389.10
NPN939	388.63
Nylon 66	393.30
PAN	373.81

**Table 3:** The glass transition temperature *T*_g_, melting point *T*_m1_ and *T*_m2_, and crystallinity *X*_c_ of nylon 66.

Separator	*T*_g_(°C)	*T*_m1_(°C)	*T*_m2_(°C)	*X*_c_(%)
NPN919	68.21	261.78	269.46	28.70
NPN929	70.04	261.80	271.78	29.11
NPN939	73.21	261.93	271.80	31.07
Nylon 66	N/A	240.00	268.00	31.33

**Table 4:** The decomposition temperature of nylon 66, PAN, and different NPN samples.

Separator	Tensile Strength (Mpa)	Elongation (%)
NPN919	49 ± 3	8 ± 3
NPN929	37 ± 1	8 ± 1
NPN939	33 ± 2	7 ± 2
Nylon 66	63 ± 7	59 ± 5

**Table 5:** The heat shrinkage properties of Celgard PP2320 and different NPN samples.

Separator	Before Heating (cm^2^)	After Heating (cm^2^)	Shrinkage (%)
PP2320	4.25	2.56	39.76
NPN919	4.11	4.04	1.70
NPN929	4.13	4.11	0.48
NPN939	4.02	4.01	0.25

**Table 6:** The porosity of Celgard PP2320 and different NPN samples.

Separator	*w*_w_ (g)	*w*_d_ (g)	Area (cm^2^)	Thickness (μm)	Porosity (%)
PP2320	0.0026	0.0096	10.67	15.1	41.78
NPN919	0.0034	0.0214	10.67	19.9	81.51
NPN929	0.0035	0.0225	10.67	20.7	82.72
NPN939	0.0036	0.0237	10.67	21.1	85.85

**Table 7:** The electrolyte uptake of Celgard PP2320 and different NPN samples.

Separator	*w*_w_ (g)	*w*_d_ (g)	Electrolyte Uptake (%)
PP2320	0.0027	0.0060	122.22
NPN919	0.0033	0.0138	318.18
NPN929	0.0035	0.0159	354.29
NPN939	0.0036	0.0182	405.56

**Table 8:** The capacitance value of Celgard PP2320 and different NPN samples.

Separator	Capacity (mAh g^−1^)
Nylon 66	121
NPN919	140
NPN929	138
NPN939	132

## Data Availability

Not applicable.
